# RS3PE syndrome developing during the course of probable toxic shock syndrome: a case report

**DOI:** 10.1186/s12879-018-3089-6

**Published:** 2018-04-13

**Authors:** Moe Kyotani, Tsuneaki Kenzaka, Ryo Nishio, Hozuka Akita

**Affiliations:** 1 Department of Internal Medicine, Hyogo Prefectural Kaibara Hospital, 5208-1, Kaibara, Kaibara-cho, Tanba, Hyogo 669-3395 Japan; 2 Division of Community Medicine and Career Development, Kobe University Graduate School of Medicine, 2-1-5, Arata-cho, Hyogo-ku, Kobe, Hyogo 652-0032 Japan

**Keywords:** RS3PE syndrome, Toxic shock syndrome, VEGF, Case report

## Abstract

**Background:**

Remitting seronegative symmetrical synovitis with pitting edema (RS3PE) is a rare syndrome characterized by “remitting,” “seronegative” (namely rheumatoid factor-negative), and “symmetrical” synovitis with pitting edema on the dorsum of the hands and feet. Recently, there have been reports that serum vascular endothelial growth factor (VEGF) is elevated in this condition.

**Case presentation:**

An 85-year-old man visited our department with a rash that had appeared 2 days earlier and a fever that had developed on the day of his visit. Based on clinical findings of fever, erythema exudativum multiforme, transitory hypotension, conjunctiva hyperemia, elevated creatine kinase, and desquamation, we suspected toxic shock syndrome (TSS). Therefore, we started treatment with vancomycin (1 g/day) and clindamycin (600 mg/day), after which his fever rapidly remitted. However, pitting edema on the dorsum of his hands and feet appeared on day 7, and the patient also had painful wrist and ankle joints. Additional tests were negative for rheumatoid factor, and anti-cyclic citrullinated protein antibodies were < 0.2 U/mL. Further, serum matrix metalloproteinase-3 (199.6 ng/mL; reference value ≤123.8 ng/mL) and serum VEGF (191 pg/mL; reference value ≤38.3 pg/mL) levels were elevated, and human leukocyte antigen-A2 was detected. The patient was thus diagnosed with RS3PE syndrome, for which he satisfied all four diagnostic criteria: 1) pitting edema in the limbs, 2) acute onset, 3) age ≥ 50 years, and 4) rheumatoid factor negativity. He was treated with oral prednisolone, resulting in the normalization of his serum VEGF level to 34.5 pg/mL 1 month after starting treatment. It is currently 1 year since disease onset, and although the patient has stopped taking prednisolone, there has been no recurrence of RS3PE syndrome.

**Conclusions:**

To the best of our knowledge, this is the first reported case of a patient developing RS3PE syndrome during the clinical course of TSS. We propose that the onset mechanism involved an increase in blood VEGF due to TSS, which induced RS3PE syndrome. As serum VEGF becomes elevated with both severe infections associated with shock and RS3PE syndrome, awareness that these conditions can occur concurrently is essential.

## Background

Remitting seronegative symmetrical synovitis with pitting edema (RS3PE) syndrome was first reported in 1985 by McCarty et al. [1]. It commonly occurs in elderly individuals and is “remitting,” “seronegative” (namely rheumatoid factor-negative), “symmetrical,” and characterized by synovitis with pitting edema on the dorsum of the hands and feet. Although its cause remains unknown, there have been recent reports of elevated serum vascular endothelial growth factor (VEGF) levels in this condition [2], suggesting a correlation between VEGF and RS3PE syndrome. VEGF is a factor involved in angiogenesis and vascular permeability [3]. It is elevated in inflammatory diseases such as rheumatoid arthritis (RA), ischemic processes such as the systemic inflammatory response, and wound healing, wherein it contributes to the restoration of tissue blood flow after an injury [3].

Elevated serum VEGF has also been linked with toxic shock syndrome (TSS) [4], a condition that results from infection by the toxin-producing *Staphylococcus aureus* or *Streptococcus* species [5]. These bacteria produce exotoxins, such as TSS toxin 1 and enterotoxin, which promote the activation of T-cells, resulting in a cytokine storm that can subsequently result in significant morbidity and mortality [5].

Here, we report for the first time a case of increased serum VEGF and development of RS3PE syndrome during the clinical course of TSS.

## Case presentation

An 85-year-old man visited our department complaining of a rash that had appeared 2 days earlier and a fever that had developed on the day of his visit. His medical history included hypertension, aortic dissection (Stanford Type B), exertional angina, and chronic obstructive pulmonary disease (COPD). He had been prescribed the following medications, which had not changed in the previous 12 months, for his medical conditions: rosuvastatin, 2.5 mg/day; ethyl icosapentate, 1800 mg/day; valsartan, 80 mg/day; amlodipine, 5 mg/day; benidipine, 8 mg/day; bisoprolol, 2.5 mg/day; lansoprazole, 15 mg/day; clopidogrel, 75 mg/day (changed to a generic brand 1 week prior to hospital presentation); sarpogrelate, 200 mg/day; and tiotropium inhalation, 18 μg/day. Two days before presentation, the patient had also been prescribed fexofenadine (120 mg/day); however, this did not result in any improvement; instead, the rash spread from the patient’s neck and right upper arm to his entire body.

**Fig. 1:**
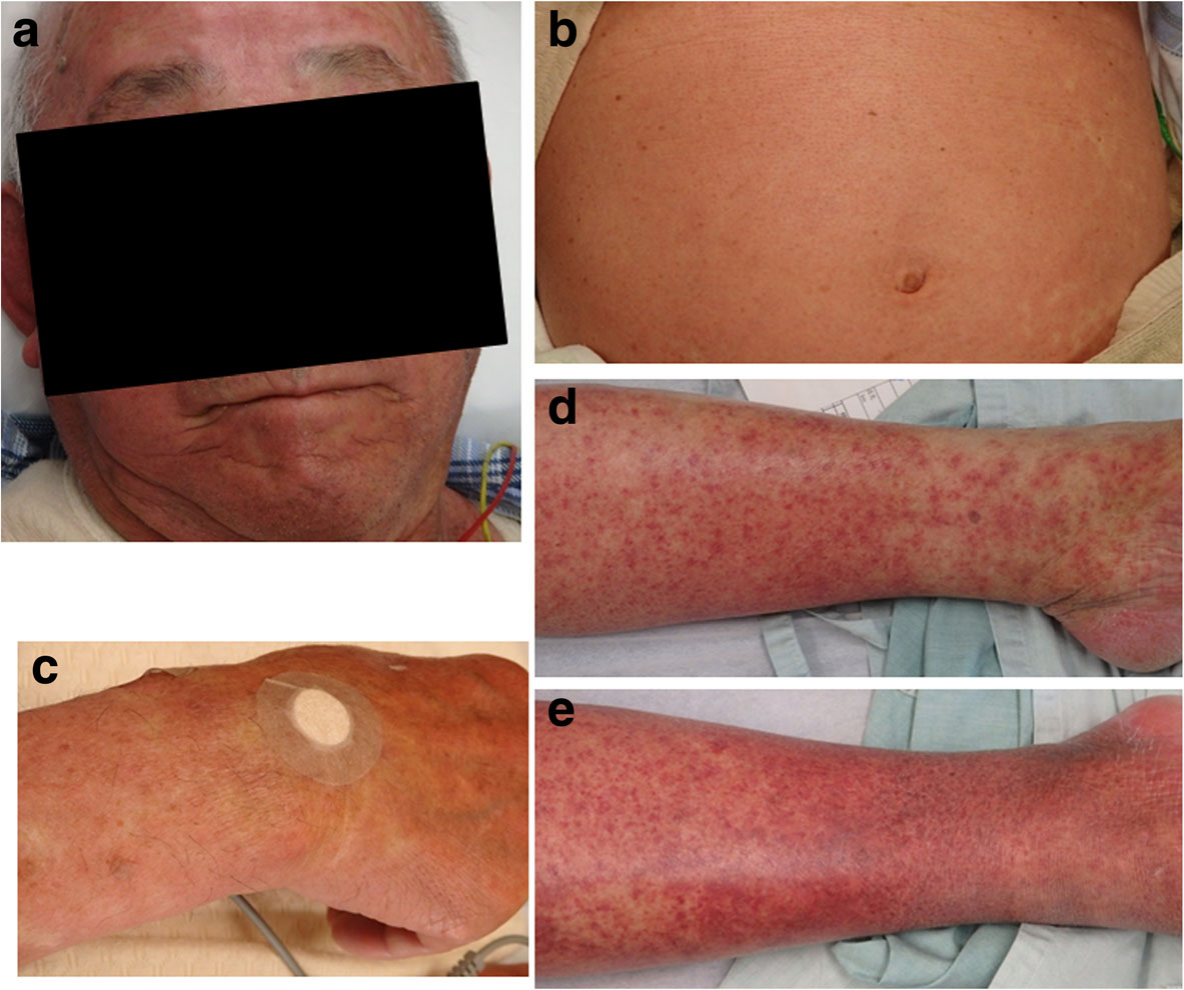
The patient had generalized erythema that was present on his face (**a**), trunk (**b**), right hand (**c**), left lower leg (**d**), and right lower leg (**e**)

Upon presentation, the patient was conscious and lucid, and his vital signs were as follows: heart rate, 90 beats/min and regular; body temperature, 39.0 °C; blood pressure, 144/86 mmHg; respiratory rate, 24 breaths/min; and peripheral oxygen saturation, 95% on room air. Physical findings included mild conjunctiva hyperemia in both eyes, and erythema on the face, trunk, and limbs (Fig. 1). The erythema was diagnosed as erythema exudativum multiforme by the dermatologist. There was no enanthema in the oral cavity and no desquamation. The superficial lymph nodes were not palpable. However, there was reddening and swelling of the right lateral malleolus. Laboratory investigation results were as follows (Table 1): white blood cell count (WBC), 19,920/μL (neutrophils, 90.9%; lymph, 4.7%; and eosinophils, 0.4%); C-reactive protein (CRP), 22.4 mg/dL; procalcitonin, 2.09 ng/mL; creatine kinase (CK), 812 U/L; blood urea nitrogen, 22.1 mg/dL; and creatinine, 1.2 mg/dL. Blood (2 sets), urine, sputum, and right lateral malleolus wound cultures were negative. Head, chest, abdominal, and pelvic computed tomography scans, as well as abdominal and cardiac ultrasounds, were also performed to find the cause of the patient’s fever, but results of these investigations were unremarkable and did not identify a source for the patient’s fever.

**Table 1:** Laboratory data on admission

Parameter	Recorded value	Standard value
White blood cell count	19.92 × 10^9^/L	4.50–7.50 × 10^9^/μL
Neutrophil	90.9%	
Lymphocyte	4.7%	
Eosinophil	0.4%	
Hemoglobin	14.7 g/dL	11.3–15.2 g/dL
Hematocrit	38.8%	36–45%
Platelet	123 × 10^9^/L	130–350 × 10^9^/L
C-reactive protein	22.4 mg/dL	≤0.60 mg/dL
Total protein	6.1 g/dL	6.9–8.4 g/dL
Albumin	3.0 g/dL	3.9–5.1 g/dL
Aspartate aminotransferase	38 U/L	11–30 U/L
Alanine aminotransferase	17 U/L	4–30 U/L
Lactate dehydrogenase	193 U/L	109–216 U/L
Creatine phosphokinase	812 U/L	40–150 U/L
Blood nitrogen urea	22.1 mg/dL	8–20 mg/dL
Creatinine	1.21 mg/dL	0.63–1.03 mg/dL
Sodium	135 mEq/L	136–148 mEq/L
Potassium	3.9 mEq/L	3.6–5.0 mEq/L
Glucose	124 mg/dL	70–109 mg/dL

After admission, we suspected that the patient’s rash was a drug eruption. The suspect drug clopidogrel was therefore switched to 100 mg/day aspirin, but the rash did not improve. Other possibilities were considered, including viral rash (herpes virus), malignant lymphoma, hemolytic streptococcal infection, and mycoplasma. However, these conditions were ruled out by various blood tests, as well as by the patient’s medical history. On day 2 in hospital, the patient developed transitory hypotension and elevated hepatic enzymes (aspartate aminotransferase, 48 U/L; and alanine aminotransferase, 50 U/L) and lactate (3.3 mmol/L; standard value: 0.56–1.39). He also developed a wheeze on the same day, which we thought was due to worsening of his underlying COPD. As such, he was treated with oral prednisolone (30 mg) for 3 days. Though this resulted in temporary improvement of his fever and a decrease in his CRP level, these variables became elevated again from day 5, and on day 7, he had desquamation on all four limbs.

**Fig. 2:**
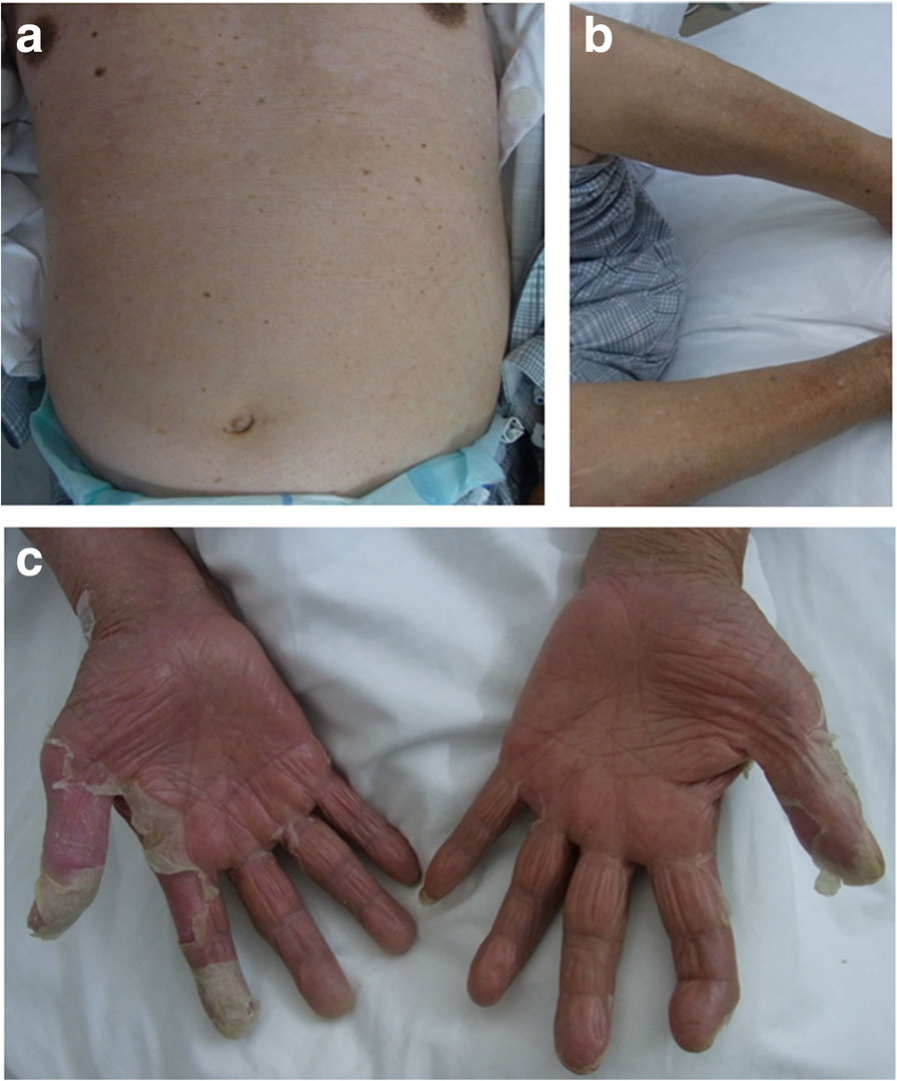
The rash on the patient’s trunk (**a**) and both lower legs (**b**) improved on day 10 of hospitalization. Membranous desquamation developed on both the patient’s hands on day 20 (**c**)

**Fig. 3:**
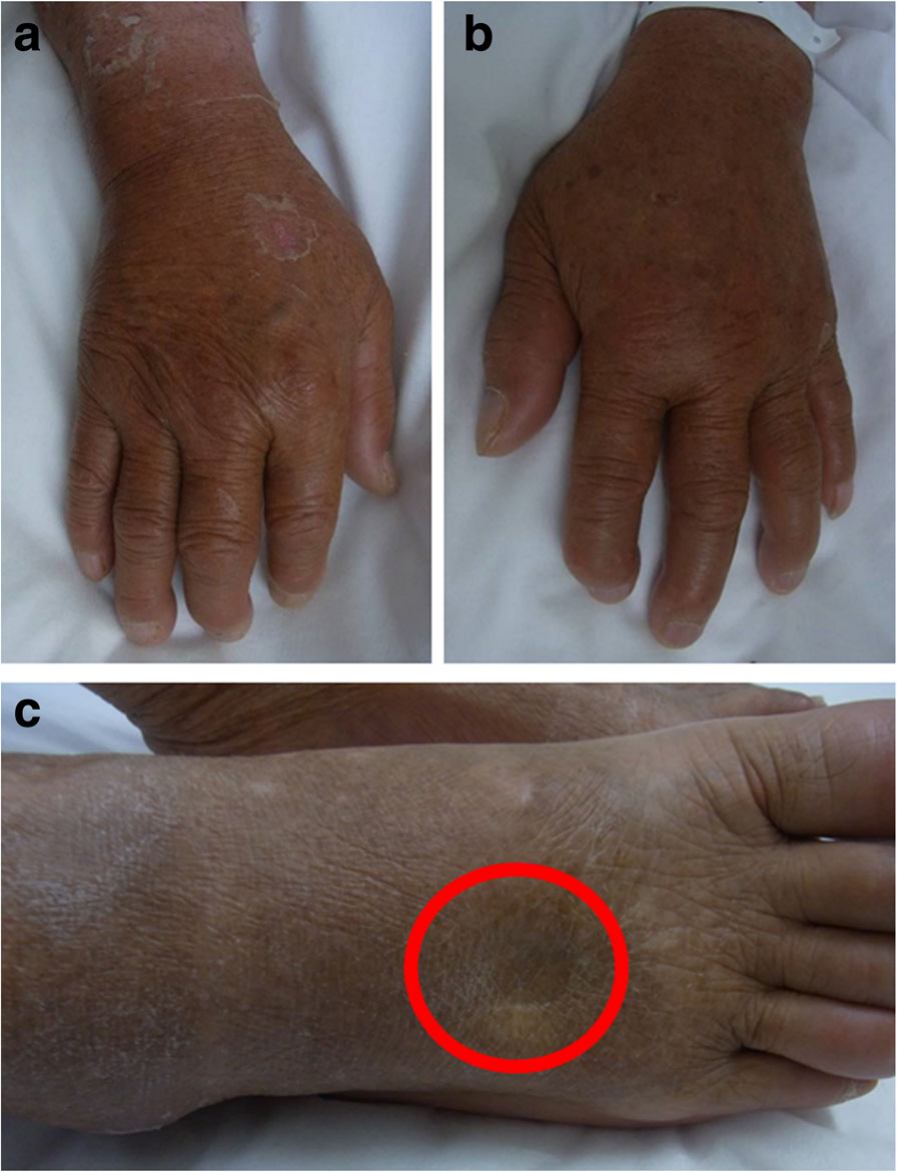
Pitting edema could be seen on the patient’s right hand (**a**), left hand (**b**), and dorsum of the right foot (**c**). Limb edema with remaining indentation is indicated by the red circle. Desquamation on part of the hand can also be seen

**Fig. 4:**
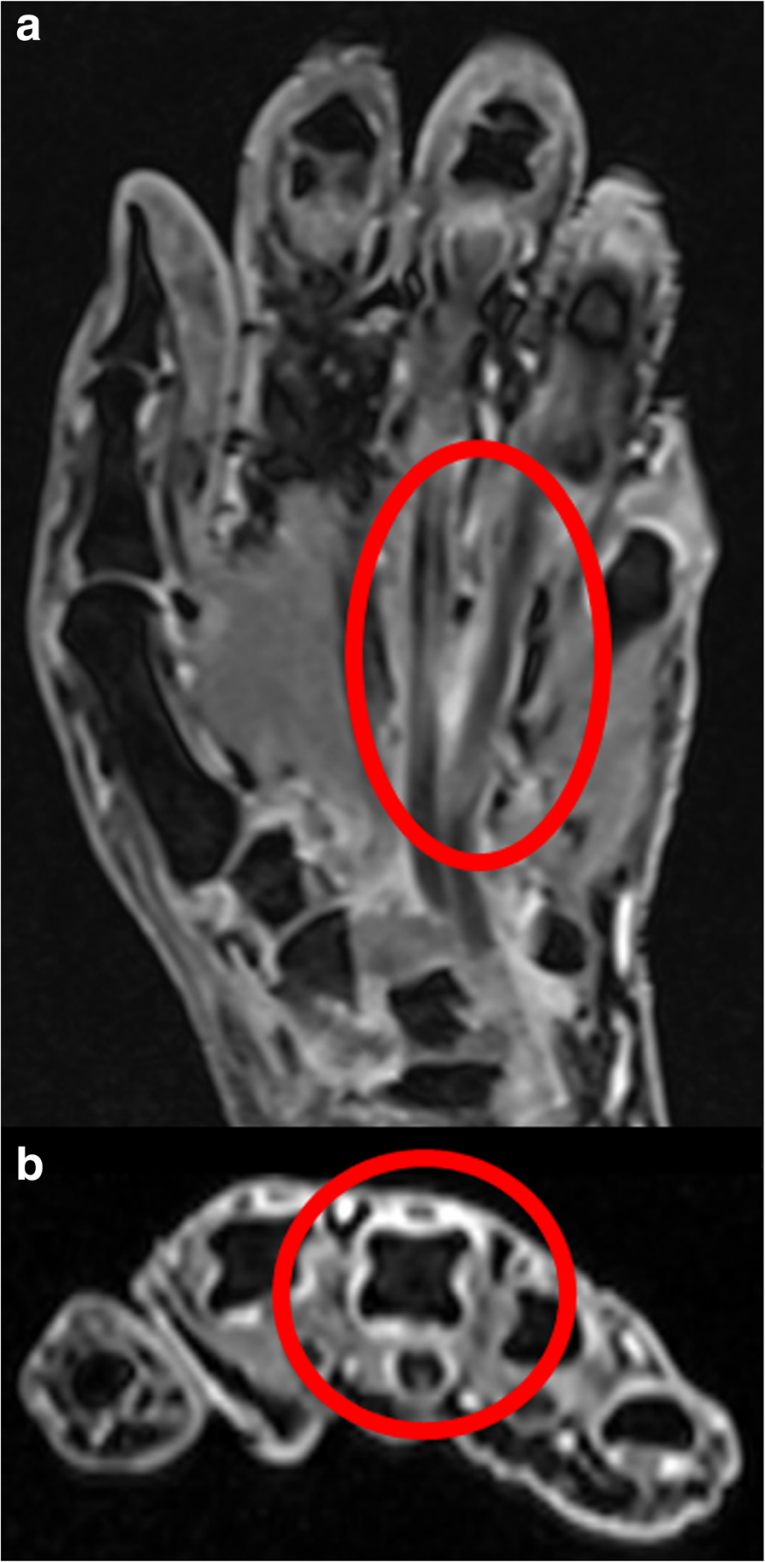
Contrast magnetic resonance imaging of the patient’s right hand. Synovial hyperplasia presented as a low intensity signal around the carpal bone (**a**) and fingers (**b**). A contrast enhancement effect was seen with gadolinium (red circles)

As no bacteria were detected from the patient’s blood culture upon admission, and based on his clinical course of fever, erythema exudativum multiforme, transitory hypotension, conjunctiva hyperemia, elevated CK, and desquamation, we suspected TSS. We therefore started treatment with vancomycin (1 g/day) and clindamycin (600 mg/day), after which his fever rapidly remitted, and his WBC and CRP improved. Four days after starting treatment with the antibiotics, the erythema on the patient’s trunk and the rash on his lower limbs disappeared. On day 20, the patient developed membranous desquamation predominantly on his fingertips, which is characteristic of TSS (Fig. 2). Pitting edema on the dorsum of his hands and feet (Fig. 3) had also appeared around the same time as the desquamation (day 7), and the patient had painful wrist and ankle joints. Additional tests were conducted to find the cause of the edema and arthralgia, but were negative for rheumatoid factor. Anti-cyclic citrullinate protein antibodies were also found to be < 0.2. Further, serum matrix metalloproteinase-3 (MMP-3, 199.6 ng/mL; reference value, ≤123.8 ng/mL) and serum VEGF (191 pg/mL; reference value, ≤38.3 pg/mL) levels were elevated, and human leukocyte antigen (HLA)-A2 was detected (Table 2). A contrast magnetic resonance imaging (MRI) scan of the patient’s painful right wrist indicated synovial hyperplasia presenting as a low intensity signal around the carpal bone, and a contrast enhancement effect was seen in the synovial membrane with gadolinium. Similar findings were seen in the metacarpophalangeal joints including the thumb and little finger, with a contrast effect seen in the synovial membrane that was suggestive of synovitis (Fig. 4).

**Table 2:** Additional laboratory data from hospital day 10, which partly informed RS3PE syndrome diagnosis

Parameter	Recorded value	Standard value
Antinuclear antibodies	160×	≤40×
Anti-citrullinated peptide antibodies	< 0.2 U/ml	< 0.2 U/ml
Rheumatoid factor	2 U/mL	2 U/mL
MMP-3	199.6 ng/mL	≤123.8 ng/mL
VEGF	191 pg/mL	≤38.3 pg/mL
HLA type	HLA-A2/A241,HLA-B51/52,HLA-Cw12/Cw14	

*MMP-3* matrix metalloproteinase-3, *VEGF* vascular endothelial growth factor, *HLA* human leukocyte antigen

**Fig. 5:**
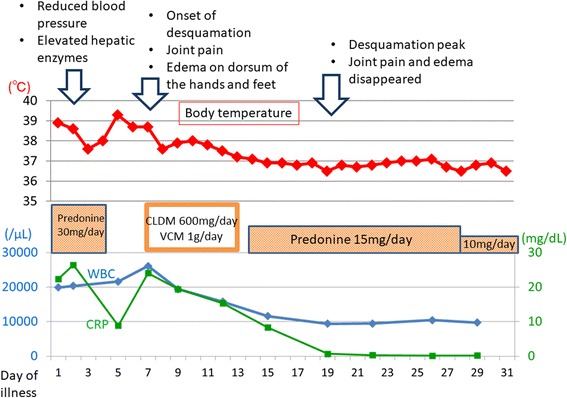
The patient’s clinical course after hospitalization. CLDM, clindamycin; CRP, C-reactive protein; VCM, vancomycin; WBC, white blood cell count

Based on these findings, this case satisfied all four diagnostic criteria for RS3PE syndrome as described by Olive et al. in 1997 [2]: 1) pitting edema in the limbs, 2) acute onset, 3) patient aged 50 years or older, and 4) rheumatoid factor negativity. The patient also satisfied the auxiliary diagnostic items of elevated MMP-3 and serum VEGF levels [3], synovial imaging on contrast MRI (synovitis findings) [5], and HLA antigen positivity. He was therefore diagnosed with RS3PE syndrome, and treated with oral prednisolone (15 mg/day) from day 14. This resulted in rapid improvement of his symptoms, and he was discharged with independent performance of activities of daily living on day 31. The patient’s clinical course during hospitalization is shown in Fig. 5.

One month after starting treatment, the patient’s serum VEGF had normalized to 34.5 pg/mL. It is currently 1 year since disease onset, and though he has stopped taking prednisolone, there has been no recurrence of RS3PE syndrome.

## Discussion and conclusions

This article introduced for the first time, to the best of our knowledge, a patient who developed RS3PE syndrome during the clinical course of TSS. With regard to TSS, two standards are widely used for its diagnosis: the US Centers for Disease Control and Prevention diagnostic criteria for TSS [6] and Tofte et al.’s diagnostic criteria for probable TSS [7] (Table 3). Our patient satisfied the six items of fever, diffuse erythema, characteristic desquamation on the extremities of the four limbs, reduced blood pressure, elevated CK, and conjunctival hyperemia, and was thus diagnosed with probable TSS. Furthermore, the patient satisfied all four criteria of the systemic inflammatory response syndrome (SIRS): fever, tachycardia, tachypnea, and leukocytosis. Though his fever, erythema, and limb desquamation rapidly improved with the administration of antibiotics, there was no apparent source of infection. Two months before consultation at our department however, the patient had pain and swelling from an insect bite on his right lateral malleolus. There was no abscess formation, and the cellulitis at this site was considered a possible infection source.

**Table 3:** Diagnostic categories of toxic shock syndrome (TSS)

Definite TSS (all criteria must be present) [6]	
Clinical Criteria	
An illness with the following clinical manifestations:	
・Fever: temperature greater than or equal to 102.0 °F (greater than or equal to 38.9 °C)	
・Rash: diffuse macular erythroderma	
・Desquamation: 1–2 weeks after onset of rash	
・Hypotension: systolic blood pressure less than or equal to 90 mmHg for adults or less than the fifth percentile by age for children aged less than 16 years	
・Multisystem involvement (three or more of the following organ systems):	
✓ Gastrointestinal: vomiting or diarrhea at onset of illness	
✓ Muscular: severe myalgia or creatine phosphokinase level at least twice the upper limit of normal	
✓ Mucous membrane: vaginal, oropharyngeal, or conjunctival hyperemia	
✓ Renal: blood urea nitrogen or creatinine at least twice the upper limit of normal for laboratory or urinary sediment with pyuria (greater than or equal to 5 leukocytes per high-power field) in the absence of urinary tract infection	
✓ Hepatic: total bilirubin, alanine aminotransferase enzyme, or aspartate aminotransferase enzyme levels at least twice the upper limit of normal for laboratory	
✓ Hematologic: platelets less than 100,000/mm^3^	
✓ Central nervous system: disorientation or alterations in consciousness without focal neurologic signs when fever and hypotension are absent	
Laboratory Criteria for Diagnosis	
Negative results on the following tests, if obtained:	
・Blood or cerebrospinal fluid cultures (blood culture may be positive for *Staphylococcus aureus*)	
・Negative serologies for Rocky Mountain spotted fever, leptospirosis, or measles	
Probable TSS (≥3 criteria and desquamation or ≥ 5 criteria without desquamation) [7]	
・Temperature ≥ 38.9 °C	
・Rash	
・Hypotension, orthostatic dizziness, or syncope	
・Myalgia	
・Vomiting, diarrhea, or both	
・Mucous membrane inflammation (conjunctivitis, pharyngitis, vaginitis)	
・Clinical or laboratory abnormalities of ≥2 organ systems	
・Reasonable evidence for absence of other etiologies	

The RS3PE syndrome that the patient developed during the clinical course of TSS is a disease concept that was first advocated by McCarty et al. in 1985 [1]. Though its diagnostic criteria have been described by Olive et al. [8] (Table 4 [9]), its onset mechanism remains unknown. It has also recently been reported that serum VEGF becomes elevated with RS3PE syndrome [2], suggesting a correlation between the condition and VEGF. Furthermore, peripheral blood VEGF is markedly higher with RS3PE syndrome than with the similar condition of RA, and can be decreased with steroid therapy. Thus, measuring serum VEGF could assist with RS3PE syndrome diagnosis [2, 10, 11]. Our case satisfied all of the RS3PE syndrome diagnostic criteria in that both MMP-3 and serum VEGF were elevated [12], synovial imaging was seen on contrast MRI (synovitis finding) [13], and the patient was positive for HLA-A2. He was also diagnosed with RS3PE syndrome based on edema and joint pain in both limbs and the marked improvement of his condition with steroid therapy.

**Table 4:** Diagnostic criteria for RS3PE syndrome [9]

Satisfies all of the following 4 items:	
・Bilateral pitting edema of both hands and/or feet (rarely unilateral hand/ ft involvement)	
・Sudden onset of polyarthritis	
・Age > 50 years (occasional reports in young people)	
・Persistent rheumatoid factor seronegativity	

In terms of the association between RS3PE syndrome and infection, tuberculosis, parvovirus B19, *Streptobacillus moniliformis*, *Escherichia coli*, *Campylobacter jejuni*, and *Mycoplasma pneumoniae* have all been listed as infections related to RS3PE syndrome [14–17]. Although there have been no reports of RS3PE syndrome developing during the clinical course of TSS thus far, elevated serum VEGF has been linked with both these conditions. In the case of infection, serum VEGF is initially mildly elevated, and becomes significantly elevated in serious conditions associated with septic shock with a concurrent reduction in VEGF receptor threshold [4]. This is thought to occur when VEGF is incorporated into the inflammatory cascade and subsequently becomes elevated through inflammation associated with severe infection, which includes ischemic processes and SIRS. This mechanism has been shown to correlate with severity scores such as the Sepsis-related Organ Failure Assessment score [4]. In our case, the patient had successive onset of the comparatively rare diseases of TSS and RS3PE syndrome. Because both are associated with elevated serum VEGF, as mentioned above, the sequential development of these conditions is not considered to be incidental. We therefore propose that serum VEGF levels are elevated with TSS as well as with hypotension-related ischemic processes, SIRS, and restoration of blood flow to the tissues after TSS, which may then have triggered RS3PE syndrome. Caution and awareness of the possibility that RS3PE syndrome may develop concurrently with severe infections associated with shock is thus essential when treating patients with conditions such as TSS.

In conclusion, to the best of our knowledge, this is the first case of a patient developing RS3PE syndrome during the clinical course of TSS. The proposed mechanism of onset is thought to have been an increase in blood VEGF due to TSS, which induced RS3PE syndrome. As serum VEGF becomes elevated with both severe infections associated with shock and RS3PE syndrome, awareness that these conditions can occur concurrently is essential.

## Abbreviations

CK: Creatine kinase
COPD: Chronic obstructive pulmonary disease
CRP: C-reactive protein
HLA: Human leukocyte antigen
MMP-3: Matrix metalloproteinase-3
MRI: Magnetic resonance imaging
POEMS: Polyneuropathy, organomegaly, endocrinopathy, monoclonal protein, skin changes
RS3PE: Remitting seronegative symmetrical synovitis with pitting edema
TSS: Toxic shock syndrome
VEGF: Vascular endothelial growth factor
